# Advances in the clinical application of ultrasound elastography in uterine imaging

**DOI:** 10.1186/s13244-022-01274-9

**Published:** 2022-09-04

**Authors:** Xia-li Wang, Shu Lin, Guo-rong Lyu

**Affiliations:** 1 Department of Ultrasound, The Second Affiliated Hospital of Fujian Medical University, No. 34 North Zhongshan Road, Quanzhou, 362000 Fujian Province China; 2 Department of Clinical Medicine, Quanzhou Medical College, Quanzhou, 362000 Fujian Province China; 3 Centre of Neurological and Metabolic Research, The Second Affiliated Hospital of Fujian Medical University, No. 34 North Zhongshan Road, Quanzhou, 362000 Fujian Province China; 4 Diabetes and Metabolism Division, Garvan Institute of Medical Research, 384 Victoria Street, Darlinghurst, Sydney, NSW 2010 Australia

**Keywords:** Elastography, Ultrasonography, Uterus, Shear wave elastography, Stiffness

## Abstract

Changes in tissue stiffness by physiological or pathological factors in tissue structure are identified earlier than their clinical features. Pathological processes such as uterine fibrosis, adenomyosis, endometrial lesions, infertility, and premature birth can manifest as tissue elasticity changes. In clinical settings, elastography techniques based on ultrasonography, optical coherence tomography, and magnetic resonance imaging are widely used for noninvasive measurement of mechanical properties in patients, providing valuable tool and information for diagnosis and treatment. Ultrasound elastography (USE) plays a critical role in obstetrics and gynecology clinical work because of its simplicity, non-invasiveness, and repeatability. This article reviews the recent progress of USE in uterine tumor diagnosis (especially early diagnosis and treatment effect evaluation), prediction of preterm birth, and intrauterine insemination. We believe that USE, especially shear wave elastography, may serve as a potential means to assess tissue stiffness, thereby improving the diagnosis and treatment of adenomyosis, fibroids, endometrial lesions, cervical cancer, and precise management of preterm birth and intrauterine insemination monitoring.

**Supplementary Information:**

The online version contains supplementary material available at 10.1186/s13244-022-01274-9.

## Key points

- The SWE is more suitable for obstetrics and gynecological applications.
- USE can assess treatment responses in uterine fibroids and adenomyosis.
- Measuring JZ through SWE could be beneficial for identifying adenomyosis.
- A risk prediction model using SWE for pre-term delivery is possible.
- Increased utilization of USE may facilitate an earlier cervical cancer diagnosis.

## Background

**Fig. 1:**
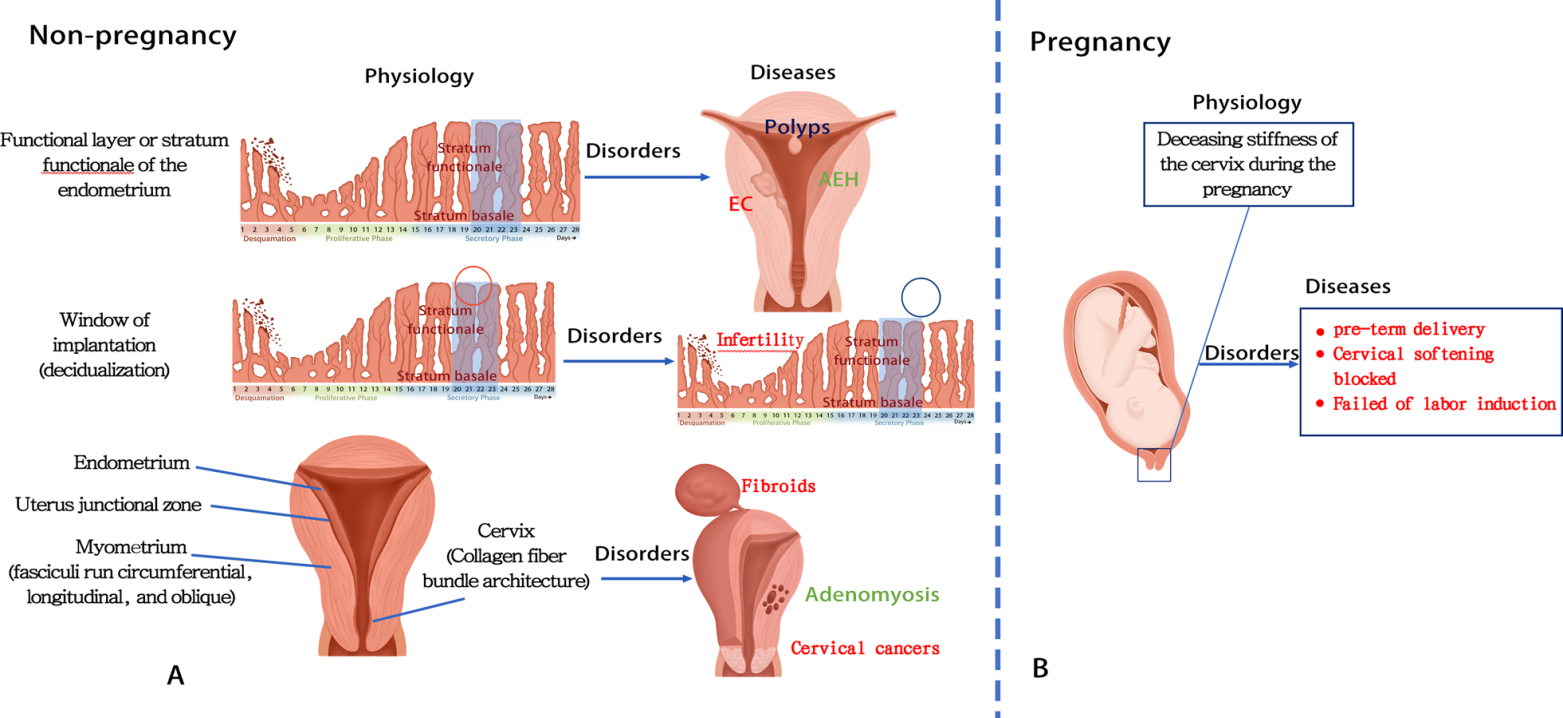
Potential involvement of stiffness in uterus disorders. **A** is for non-gestation period while **B** is for gestational period. The stiffness of uterus fibroids, EC, cervical cancers, infertility, and disorders in the cervix during pregnancy increases than normal tissues (Red font), as well as endometrium polyps decrease (blue font). AEH and adenomyosis are still uncertain (green font). *AEH* atypical endometrial hyperplasia, *EC* endometrium cancer

The female reproductive system is a complex multi-organ system with multiple closely regulated functional processes [1]. Therefore, uterine stiffness is one of the important mechanical parameters and physical properties of uterine tissue and is closely related to the biological characteristics of the uterus [2]. Different cycles of uterine tissue, such as proliferative or secretory, or gestational and non-pregnant, have different degrees of stiffness [3]. In addition, some pathological processes may manifest as changes in the elasticity of uterine tissue [4]. For example, compared with normal myometrium, uterine fibroids are characterized by altered mechanical homeostasis and increased stiffness due to excess extracellular matrix [5]. Adenomyosis is usually diagnosed as myometrial glandular and interstitial heterotopia. Histopathology shows hyperplasia and hypertrophy of surrounding smooth muscle cells with hyper-fascicular trabecular pattern and increased extensive fibrosis and micro-vascularization [6, 7]. Benign lesions such as endometrial hyperplasia, polyps, and endometrial atrophy originate from endometrial soft tissue and endometrial gland hyperplasia, containing a small amount of fibrous interstitial components, have soft stiffness, and are accompanied by an increased proportion of nucleosomes. Therefore, malignant transformation may be associated with increased stiffness [8]. Given that, studying the stiffness of tumor tissue gives a deep insight into its characteristics and behavior (Fig. 1a).

Furthermore, these physiological changes lead to biomechanical modifications in uterine tissue [9]. Changes in the collagen content and structure of uterine tissue during pregnancy lead to uterine tissue physiological remodeling and tissue elasticity [10]. The collagen and elastic fiber structure of the cervix undergoes rapid and dramatic changes to fulfill its different physiological roles for competence during pregnancy and compliance during birth [11]. Moreover, elastography changes due to pregnancy complications or abnormal delivery have contributed to cervical softening disorders (Fig. 1b) [12]. Therefore, assessing cervical elasticity to predict premature delivery and labor induction outcomes may influence the choice of clinical treatment.

The endometrium undergoes a receptive period during the menstrual cycle where blastocysts can invade.This period is defined as the “window of implantation” and is of limited duration [13]. Precise determination of the window of implantation can significantly improve the efficacy of assisted reproductive technology (ART) [14]. It is well established that endometrial elastography reflects biochemical and molecular changes in the endometrium throughout the menstrual cycle [15]. Concurrently, transvaginal ultrasound is widely used and offers a good opportunity for rapid and accurate assessment of the endometrium. However, the clinical relevance of ultrasonographic markers remains uncertain and further studies are needed to conclude [16]. Herein, we sought to review the potential ability of USE to predict pregnancy rates following intrauterine insemination (IUI) cycles.

Ultrasound elastography has also been widely used to diagnose various organs disorder such as the liver, breast, thyroid, and blood vessels [17]. This promising technique has played an important role in obstetrics and gynecology due to its simplicity, non-invasiveness, and reproducibility [18]. This article reviews the recent advances in USE application for diagnosing myometrium, endometrial and cervical tumors, especially the evaluation of early diagnosis and treatment. In pregnancy, our review focuses on improving the efficiency of predicting preterm birth and identifying a potential approach to precisely manage neonatal respiratory complications. In addition, the use of USE to monitor IUI can also be discussed concurrently.

## Principles of ultrasound elastography in uterine diseases

**Fig. 2:**
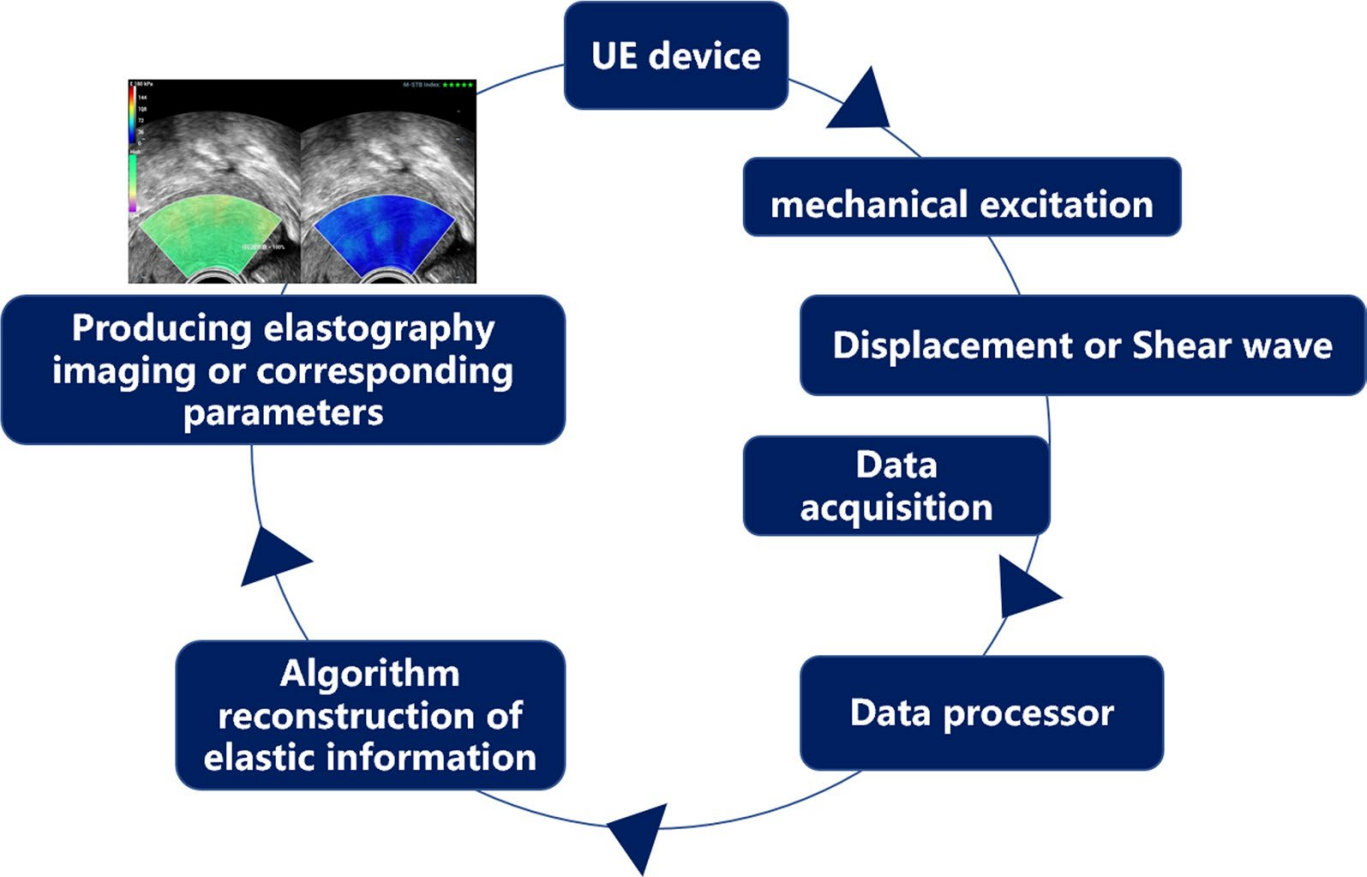
Flow chart of ultrasound elastography

Ultrasound is the most commonly used imaging diagnostic tool in obstetrics and gynecology; however, ultrasound imaging also has some disadvantages, such as low contrast between abnormal tissue and surrounding tissue. Relying on operator subjectivity and subsequent inability to distinguish the mechanical properties of tissues with the same ultrasonic echogenicity is also a disadvantage of ultrasound imaging [19]. Notably, elastography techniques can display elastic tissue changes due to specific pathological or physiological processes [20]. All elasticity measurement and imaging methods typically introduce a mechanical excitation and monitor the resulting tissue response. The different techniques currently available USE techniques can be divided into strain imaging and shear wave imaging (SWI) according to the measured quantity [21]. The workflow of USE can be simplified as follows: First mechanical excitation is applied to the target tissue, and then, the displacement or shear wave generated by the target tissue is obtained. Finally, the different signals are encoded and imaged, or corresponding parameters are measured [22] (Fig. 2). Strain and SWI require mechanical excitation, which can be divided into (A) manual compression (by hand or using cardiovascular pulsation or respiratory motion), (B) acoustic radiation force pulse (ARFI), and (C) external mechanical vibration [23]. Currently, the clinical imaging diagnostic methods mainly include strain elastography (SE), transient elastography (TE), ARFI imaging (ARFI imaging), shear wave speed measurement, and imaging using acoustic radiation force impulse excitation [24].

Strain imaging should measure the “stress” applied to organizational structure relative to the resulting “strain” or deformation. SE and ARFI imaging belong to this category, and SE is the most widely used mode in obstetrics and gynecology. In SE, stimulation methods include manual tissue compression by the operator using an ultrasonic transducer or generated internally by physiological movements, such as the cardiovascular or respiratory systems. Transparent color overlay on B-mode images is used for visualization, and strain-based elastography is generated to transform tissue strain information into two-dimensional grayscale or pseudo-color images, which become strain profiles [25]. It is worth noting that the color scale may vary by ultrasound provider. The strain ratio (SR), which is the ratio of strain measured in a target lesion region of interest (ROI) to strain measured in adjacent (usually normal) reference tissue ROI, indicating that the SR is higher and the target lesion compresses much more difficult, and then, the stiffness is greater, and vice versa. However, artificial or physiological pressures cannot be quantified, requiring operator skills and experience for promising results.

**Fig. 3:**
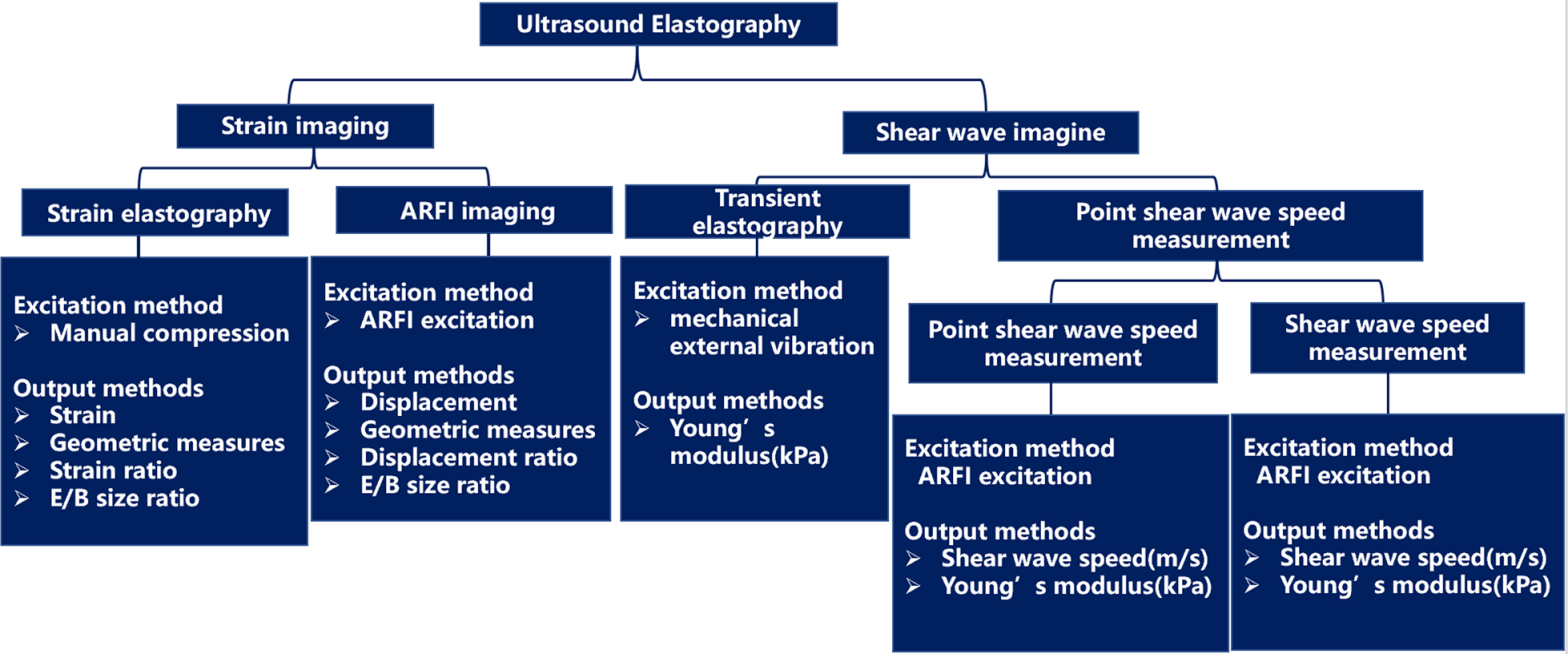
The excitation and output methods for different ultrasound elastography modalities. *ARFI* acoustic radiation force impulse

SWI utilizes dynamic pressure to generate shear waves in parallel or vertical dimensions. Shear wave velocity can qualitatively and quantitatively estimate tissue elasticity [26]. The process can be summarized as follows: 1) The focused acoustic radiation force pushes the short-duration pulse; 2) the shear wave is generated within the organ of interest; 3) the speed of the shear wave propagation is measured away from the push position; and 4) the reported information can be averaged within an ROI (a point measurement) or as an image (shear wave elastography) and values are reported as shear wave velocity (Cs) or converted to the elastic modulus. The output obtained from each elastography technique corresponds to the measured physical quantity, as shown in Fig. 3.

Shear wave elastography (SWE) is the most widely used SWI in obstetrics and gynecology among different kinds of SWI [27]. The uterus is an active pelvic organ. Therefore, it is challenging to control the artificial pressure consistently to ensure repeatability when using SE. Meanwhile, SE has the limitation of difficulty in imaging deep pathological tissues, so this technology is mostly used to detect direct contact organs, such as the elasticity detection of superficial organs. The SWE can theoretically detect depths up to 8 cm without operator pressure and has quantitative properties, making it more suitable for obstetrics and gynecology applications.

## Ultrasound elastography and different uterine diseases

Ultrasound elastography, especially shear wave elastography, has emerged to assess tissue stiffness in recent years, thereby improving the diagnosis and treatment of clinical uterine fibroids, endometriomas, cervical tumors, etc.

## Normal myometrium, uterine fibroids, and adenomyosis

**Fig. 4:**
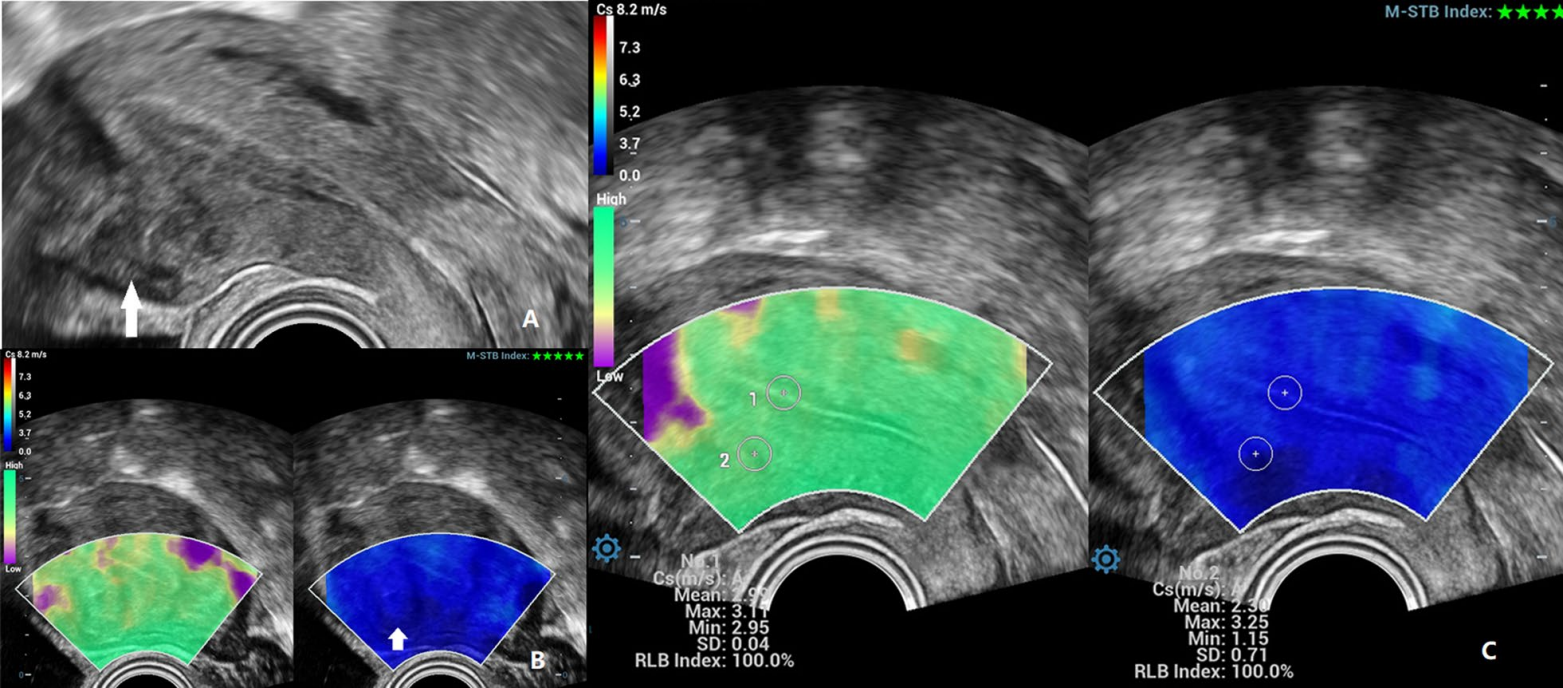
SWE used to diagnose of uterus fibroids. **A** Transvaginal ultrasound showed a hypoechoic lesion in the anterior inferior uterine segment (marked with a white arrow). **B** SWE showed a lighter blue color pseudocapsule that circling around the fibroid (marked with a white arrow). **C** Locating the region of interest at the lesion 2 and shear wave speed (Cs) measured automatically

According to the physical characteristics of uterine fibroids, the stiffness of uterine fibroids should be greater than the surrounding myometrium. This result is also supported by the current ultrasound elastography study of uterine fibroids (Fig. 4), with SWE showing images measuring uterine fibrosis [28–30]. However, elastography stiffness is controversial in assessing adenomyosis. (Table 1 reviews the literature on USE in diagnosing normal myometrium, uterine fibroids, and adenomyosis.) Frank et al. obtained elastography data from 206 uteri with SE and maximum SR (ROI lesions/ROI healthy tissue). They demonstrated that the maximum SR values for uterus fibroids were 2.65 [2.12; 3.34] and 0.44 [0.36; 0.46] for adenomyosis. The SR of uterine fibroids was greater than 1, and the SR of adenomyosis was less than 1, indicating that uterine fibroids were stiffer than normal tissue, and adenomyosis was softer. They further suggested that SE can help differentiate uterine fibrosis from adenomyosis [31]. However, Liu et al. also used SE to evaluate the stiffness of adenomyosis and uterine fibrosis, and the results showed that the stiffness of adenomyosis lesions was significantly higher than the normal uterus (*p* < 0.0001) and even higher than that of fibroid lesions (*p* = 0.006). This study further found that lesion stiffness was positively correlated with fibrosis degree, negatively correlated with E-cadherin and progesterone receptor expression levels, and positively correlated with dysmenorrhea severity and the number of menses. SE can guide the choice of the best treatment modality for patients [32]. The results on adenomyosis stiffness in these two studies were opposite, probably because SE was affected by probe pressure, ROI selection was subjective, and AM lesions generally did not have obvious border shifts on ultrasound or SE.

**Table 1:** Overview of the studies on USE in diagnosing UF and UM

Year	Authors	Patient numbers and type of lesions	Type of elastography	Type of study	Diagnostic parameters	Diagnostic performance or research results
*Assessment of the normal uterus*						
2019	Manchanda et al. [58]	NM = 56	SWE	Prospective cohort study	E mean	The E mean was 25.54 ± 8.56 (endometrium), 40.24 ± 8.59 (myometrium), and 18.90 ± 4.22 (cervix). There was no significant difference in E mean for women in different menstrual phases (*p* = .176) or in different age groups (*p* = .376)
2015	Soliman et al. [57]	NM = 32	ARFI	Prospective observational study	Cs mean	The menopausal status did not have any significant influence on the Cs measurements. The Cs means were 2.05 ± 0.77 m/s (endometrium) while 2.82 ± 0.77 m/s(myometrium)
*Lesions of the uterus*						
2022	Pongpunprut et al. [33]	NM = 25, UF = 25, AM = 25	SWE	Prospective cross-Sectional Study	Cs mean	The Cs differed between NM and AM (*p* = 0.019) with the cut-off point at 3.465 m/s and 80% sensitivity, 80% specificity, and AUC of 0.80 (95% CI 0.68–0.93) (*p* < 0.001). SWE could not differentiate AM from UF or UF from NM
2021	Görgülü et al. [34]	UF = 98, AM = 37 NM = 40	SWE, SE and MRI ADC	Retrospectively case–control study	SR mean, SR max, ADC values, Cs mean, and Cs max	SE, SWE, and MRI ADC could be useful in differentiating UF and AM (*p* < 0.001 for all three), and none of these methods were statistically superior to each other in differentiating the UF from the AM (*p* < 0.001)
2019	Zhang et al. [49]	NM = 16, UF = 12, AM = 6	SWE	Prospective case–control study	Cs mean	Cs mean in NM was 4.861.9 m/s, compared with 4.962.5 m/s in AM and 5.662.5 m/s in UF (*p* = 0.34). SWV for AM and UF did not differ significantly (*p* = 0.40)
2018	Bildaci et al. [29]	AM = 28, NM = 62	vitro ARFI	Prospective case–control study	Cs mean	The Cs mean of AM (4.22 ± 1.62 m/s) showed a significant difference compared to that of NM (3.22 ± 0.90 m/s) (*p* < 0.01)
2018	Stoelinga et al. [30]	NM = 10, UF = 10, AM = 10	SE	Prospective diagnostic study	Uterine volume for AM and fibroid volume for AF	The sensitivity of SE in the diagnosis of UF and AM was 82% and 91%, and the specificity was 95% and 97% with high inter-observer and inter-method agreement
2018	Liu et al. [32]	NM = 141, UF = 75, AM = 147	SE	Prospective control study	SR mean, SR max, SR min	The stiffness of AM lesions was significantly higher than that of UF (*p* < 0.01)
2016	Frank et al. [31]	NM = 143, UF = 41, AM = 22	SE	Prospective case–control study	SR max: stored as the “lesion index”	“Lesion indices” of UF, AM, and NM were 2.65, 0.44, and 1.19, respectively, and were significantly different between them (*p* < 0.001)
*Assessment of treatment*						
2020	Samanci et al. [36]	UF = 33	SWE	Prospective case–control study	Cs mean	The post-UAE Cs mean of UF (3.34 ± 3.9 kPa) was significantly lower than that of the pre-UAE (17.16 ± 4.8 kPa) (*p* < 0.001). There was excellent agreement between the 2 blinded observers in Cs mean
2019	Xie et al. [35]	AM = 45	SE	Prospective case–control study	scoring system	In 12 cases who were pregnancy during the follow-up, the mean elasticity score was significantly higher for the uterine after therapy than before (3.6 ± 0.3 vs 2.3 ± 0.5, *p* = 0.004)

*USE* ultrasound elastography, *NM* normal myometrium, *SE* strain elastography, *SWE* shear wave elastography, *E* Young’s modulus, *Cs* shear wave speed, *MRI ADC* magnetic resonance imaging apparent diffusion coefficient values, *UF* uterine fibroids, *UAE* uterine artery embolization, *AUC* area under the curve, *ARFI* acoustic radiation force imagine, *AM* adenomyosis, *SR* mean strain ratio mean, *SR* max strain ratio maximum, *SR* min strain ratio minimum. References were presented in Supplementary text

Another controversial point is whether USE can differentiate adenomyosis from uterine fibroids. Zhang et al. applied SWE to evaluate uterine adenomyosis and uterine fibrosis. They reported a Cs of 4.861.9 m/s in normal myometrium, 4.962.5 m/s in adenomyosis, and 5.662.5 m/s in fibrosis, with no significant difference in Cs between adenomyosis and fibrosis (*p* = 0.40) [31]. Pongpunprut et al. also demonstrated that SWE could differentiate adenomyosis from the normal uterus, but there was no significant difference in Cs between adenomyosis and fibroids groups [33]. Görgülü et al. reported that both SE and SWE were used to differentiate leiomyomas from adenomyosis, and both SE and SWE were statistically different (*p* < 0.001) [34]. It was proposed that there are contradictory results because of the limited number of studies hitherto performed with SE or SWE, and studies with larger patient groups are required.

Although controversial, both SE and SWE have been shown to differentiate between normal muscle layers, adenomyosis, and uterine fibroids, so SE may help assess response to therapy. Xie et al. investigated the effect of GnRH agonist (GnRHa) on adenomyosis by SE. They found increased elasticity in adenomyosis after GnRHa treatment, associated with spontaneous pregnancy in infertile patients [35]. Using SWE to study the response of uterine fibrosis patients to uterine artery embolization (UAE), Samanci et al. found significantly lower uterine fibrosis values after uterine artery embolization than in normal tissue. SWE can be used as a follow-up tool for uterine fibrosis after UAE [36].

**Fig. 5:**
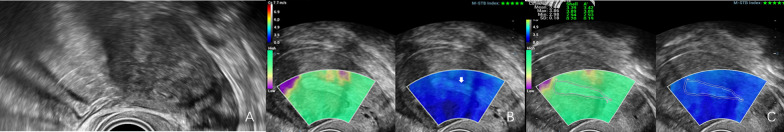
SWE used to display of uterus junctional zone (JZ). **A** Transvaginal grayscale ultrasound showed JZ appeared as a fuzzy region. **B** JZ in SWE can be seen clearly (marked with a white arrow) and distinguished from the surrounding healthy tissue. **C** The endometrium is delineated, and then, the JZ is delineated by shell function key and the shear wave speed (Cs) of both regions can be obtained simultaneously

Adenomyosis severely affects the quality of life of patients [37]. However, the stiffness changes in adenomyosis are unclear. Recently, the uterine junctional zone (JZ) has been defined as the inner 1/3 of the myometrium between the endometrium and the myometrium. Its structural and functional disturbance has been reported to be involved in the occurrence and development of adenomyosis [38]. In 2021, a consensus was reached on a revised definition of the Morphological Uterine Ultrasound Assessment (MUSA) features of adenomyosis, which considered irregular union bands as an indirect feature of adenomyosis [39]. Since adenomyotic lesions near the JZ may have more advanced fibrosis than newer lesions at the mid-uterine wall, different measurement locations have different Cs values [40]. Therefore, we hypothesized that measuring the Cs of JZ could improve the accuracy of SWE in identifying adenomyosis. Figure 5 shows the procedure of JZ displayed and measured by SWE.

In summary, USE can be used as an alternative diagnostic tool to differentiate between normal myometrium and uterine fibroids, and normal myometrium and adenomyosis, suggesting a potential role for USE in assessing treatment response. Whether USE can distinguish uterine fibroids from adenomyosis is still controversial.

## Endometrial tumors

The USE study in endometrial tumors is still in its infancy, and the literature is limited [41]. Czuczwar et al. demonstrated that SE could not be used to screen intrauterine lesions. However, SE can show the different stiffness of endometrial polyps and submucosal fibroids when the lesions are already visible on B-mode sonography [42]. Du et al. explored the diagnostic value of transvaginal SWE for endometrial polyps, endometrial hyperplasia, and endometrial cancer and found that the maximum value of Young' modulus (E) was 27.28 ± 10.28 kPa in endometrial polyps, 36.32 ± 15.04 kPa in the endometrial hyperplasia cases, and 86.66 ± 42 kPa in the endometrial cancer cases (*p* < 0.05). SWE can be used as an auxiliary method for diagnosing and differential diagnosis of endometrial cancer [43]. Ma et al. further evaluated the diagnostic value of SWE for endometrial cancer and atypical endometrial hyperplasia (AEH). They established a predictive logistic regression model to diagnose endometrial cancer and AEH, suggesting that SWE can further diagnose endometrial cancer and AEH [44]. However, Vora et al. found no statistical difference in elasticity between carcinoma and AEH (*p* = 0.19) [45]. In a later study, the researchers measured the elasticity ratio of endometrial lesions to the myometrium (E/M ratio), arguing that using the myometrium as an internal control would more objectively describe mass lesions. The inconsistency in the parameters they used may be the reason for the contradictory results of the two studies. (Table 2 lists studies of USE in the diagnosis of endometrial lesions.) Notably, there is anisotropy in the uterine myometrium, and we believe that the index Cs, rather than E, is more suitable to assess the stiffness ratio of the endometrium to the myometrium. Zhao et al. reported that the determination of endometrial cancer by SWE can determine whether it has invaded the myometrium and the depth of myometrial invasion, which can clinically determine the surgical method and determine the prognosis [46]. Although there are limited studies, the accuracy of SWE in diagnosing endometrial disease is outstanding. Given its usefulness, we speculate that future studies may focus on the ability of SWE to assess the depth of invasion and staging of endometrial cancer. More quantitative indicators, combined with clinical symptoms, are helpful for diagnosis.

**Table 2:** Overview of the studies on USE for endometrium diseases

Year	Authors	Patient numbers and type of lesions	Type of elastography	Type of study	Diagnostic parameters	Diagnostic performance or research results
*Endometrium tumors*						
2022	Vora et al. [45]	AEH = 11, EC = 29, Submucosal UF = 13, endometrial polyp = 14, Focal AM = 7	SWE	Prospective control study	E, E/M ratio	The elasticity of five pathologies was significant difference (*p* < 0.001). E mean of endometrial polyp was lowest (*p* < 0.01), and no significant difference was noted in E mean of EC and AEH (*p* = 0.19)
2021	Ma et al. [44]	benign lesions = 85 and EC including AEH = 37	SWE	Prospective case–control study	E max, E mean	E max and E mean were identified as independent risk factors for EC and AEH
2021	Du et al. [43]	Endometrial polyps = 45, AEH = 29 and EC = 66	SWE	Prospective diagnostic study	E mean, E max, and E min	E max has the highest diagnostic value with the truncation values of 52.45 kPa to distinguish between normal endometrium and EC
2016	Gultekin et al. [41]	AEH = 22, endometrial polyps = 20, and NU = 64	SE	Prospective control study	B/A ratio	AEH and endometrial polyps had significantly lower B/A ratios than NU (*p* < 0.01); however, there is no significant difference between them (*p* > 0.05)
2016	Czuczwar et al. [42]	endometrial polyps = 29 and submucosal fibroids = 18	SE	Prospective diagnostic study	Elastographic color map	The accuracy for SE in distinguishing endometrial polyps and submucosal fibroids was 89.4% and had the highest proportion of correct findings(*p* < 0.001)
*Infertility*						
2021	Kabukçu et al. [62]	197 IUI cycles (148 infertility women)	SE	Prospective diagnostic study	SR (endometrium/parametrial tissue)	The SR was not different between pregnant and non-pregnant groups (*p* = 0.651). SR was not predictive for pregnancy
2021	Shui et al. [63]	117 of infertility and 35 of pregnancy	SWE	Prospective diagnostic study	SR (endometrial/subendometrial areas)	The AUC up to 0.949 for predicting pregnancy by using age and ultrasonographic factors including uterine peristalsis, uterine spiral artery, and SR. The sensitivity was 0.83, and specificity was 0.96
2017	Swierkowski-Blanchard et al. [61]	100 women for IUI	SE	Prospective diagnostic study	SR	The SR was significantly higher (2.4 ± 1.3 vs. 1.5 ± 0.7, *p* < 0.001) in future pregnant women

*USE* ultrasound elastography, *EC* endometrial carcinoma, *AEH* atypical endometrial hyperplasia, *UF* uterine fibroids, *E/M ratio* the ratio of mean elasticity of the endometrial lesion to myometrial elasticity, *SE* strain elastography, *SWE* shear wave elastography, *SR* strain ratio, *E* Young’s modulus, *E max* Young’s modulus maximum, *E mean* Young’s modulus mean, *B/A* ratio the ratio of mean elasticity of the endometrium to adjacent myometrium, *AUC* area under the curve, *IUI* intrauterine insemination. References were presented in Supplementary text

## Cervical tumors

Cervical cancer (CC) is only cancer with clinical staging in gynecology. According to FIGO, staging is the key to selecting treatment methods. SE and SWE have been used for the differential diagnosis of CC and to assess the degree of invasion [47]. Fu et al. studied SWE in CC (*n* = 40), benign cervical lesions (*n* = 40), and 40 healthy volunteers, and the results showed that the mean Cs of cervical cancer patients were significantly higher than benign cervical lesions and normal cervix (*p* < 0.05). The results showed that SWE was more accurate than b-ultrasound in evaluating vaginal fornix and uterine infiltration (*p* < 0.05) [46]. Furthermore, SWE was evaluated for uterine and vaginal fornix invasion, and the results showed that SWE was more accurate in assessing vaginal fornix and uterine invasion than B-mode sonography only (*p* < 0.05) [48].

USE may have an important role in the early evaluation of chemotherapy or radiation therapy treatment efficacy in CC. Zhang et al. performed SE examination in 160 patients with suspected CC and compared the results with the pathological and clinical stages of CC. Radiotherapy was used for patients confirmed as CC75 in 160 suspected CC patients. The results demonstrated that SE has a certain clinical value in the diagnosis and efficacy evaluation of CC, and its sensitivity (94.67%), specificity (92.94%), and diagnostic accordance rate (93.75%) [49]. In 2021, Shao et al. conducted a systematic review of the UE application in CC and concluded that both SE and SWE might have important roles in the differential diagnosis of CC, assessment of the degree of invasion, clinical staging, and early evaluation of treatment effects [50].

It is well established that SE provides semiquantitative results, while SWE provides quantitative results, expressed in m/s or kPa, making it difficult to compare SE and SWE when analyzing CC. Technologically, SWE is superior to SE due to its ability to evaluate the anisotropic elasticity and viscosity of cervical lesions, which may help improve diagnostic performance and open doors for new clinical applications [51].

Given the viral etiology and its sexual transmission, cervical intraepithelial neoplasia (CIN) occurs mainly in young patients of reproductive age, who want to preserve their fertility [52]. In 2021, Dudia-Simon et al. revised the literature on the role of elastography in CC and CIN, from diagnosis and staging to predicting the response to oncologic treatment. In the meta-analysis, they share consistent opinions with Shao's review that USE can be used to assess normal cervical variants and positive diagnosis of CC, clinical staging, and the prediction of therapeutic response in CC. However, they argue that the method used to distinguish CC and CIN is not applicable [53]. CIN is a precursor of CC and has less pathological changes than CC. There is no unique feature in USE to detect CIN due to image noise, reduced resolution, and unclear image edge recognition [54]. Sun et al. introduced a denoising algorithm for an intelligent bilateral filter, which has improved image quality when used in applications. Combined with human papillomavirus (HPV) testing to diagnose CIN, the results showed that the accuracy, sensitivity, and specificity of this new technology were 95%, 95%, and 98%, respectively [55]. In summary, the bilateral filter intelligent denoising algorithm has a good denoising effect on ultrasonic elastography. The USE images processed by the algorithm combined with HPV detection have a better diagnostic effect on CIN.

## Infertility

**Fig. 6:**
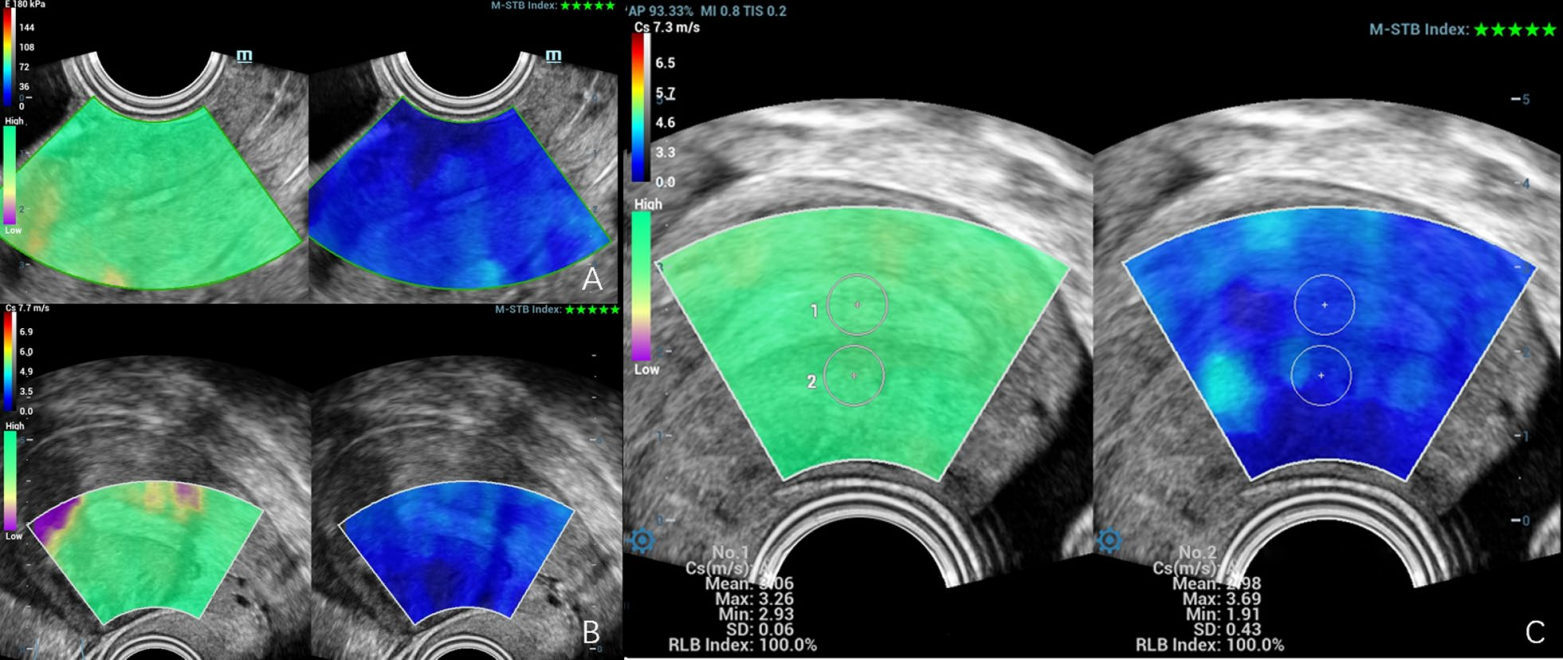
SWE for normal endometrium. SWE showed a relatively uniform blue area in the proliferative endometrium (**A**) and secretory endometrium (**B**). Image **C** further showed that the region of interest was selected in endometrium 1 and myometrium 2 and that shear wave speed (Cs) were acquired

During the menstrual cycle, major structural changes occur in the endometrium. When desquamated, the upper, functional layer of the endometrium is completely sloughed off, followed by reconstruction during the proliferative phase and then the secretory phase [56]. Soliman et al. showed that menopausal status did not significantly affect the Cs measurements by ARFI [57]. In 2019, Manchanda et al. found that there was also no significant difference in mean endometrial elasticity values in women at different physiological stages (*p* = 0.176) or in different age groups (*p* = 0.376) when using SWE (Fig. 6 shows the elasticity imaging and measurement of normal endometrium through SWE. Table 1 lists the studies on USE in the assessment of normal endometrium) [58]. In addition, three-dimensional multi-frequency magnetic resonance elastography (MRE) combined with a multi-frequency dual-elastic visco-inversion method was used to measure the response of viscoelastic materials to vibration. The results showed that the complex shear modulus |G *| and the |G *| of the endometrium were higher during the proliferative phase (3.34 ± 0.42 kPa) than during the early secretory phase (1.97 ± 0.34 kPa) in healthy volunteers [59]. However, whether these differences reflect overall differences in the entire endometrium or between functional and basal endometrial layers is uncertain. MRE uses the magnitude of the complex shear modulus G, which contains both elastic and viscous components and is calculated from phase-contrast multiphase pulse sequence data, while SWI measures E or Cs [60]. Estimations of these values depend on the used frequency of excitation, making a comparison of E or Cs reported in USE and G in MRE is challenging [60]. Considering that the connective tissue surrounding the extensive functional glands is very loose, this contributes to the increased softness during the secretory phase. MRE is costly and time-consuming; therefore, a multicenter study with a larger sample size using the same elastography technology and vendor is worth further verifying whether SWE has significant differences in endometrial elasticity values in women with different menstrual periods.

The endometrium lines the uterine cavity, implants the embryo, and provides the environment for the embryo to develop and grow. Swierkowski-Blanchard et al. assessed endometrial elasticity (using SR) before IUI and showed significantly higher SR (with stiffer myometrium) [61]. SE provides a promising and innovative tool for IUI monitoring. For abnormal elasticity, appropriate strategies (another IUI with specific treatments, in vitro fertilization, etc.) should be assessed to improve fertility outcomes. However, Kabukçu et al. found that endometrial SR had no significant effect on pregnancy rate during gonadotropin-stimulated artificial insemination cycles. It appears that SR does not predict IUI outcomes [62]. Currently, the efficiency of ultrasonic detection of endometrial receptivity is still inconclusive, and we believe that single parameters are unreliable in predicting pregnancy outcomes. Shui et al. obtained endometrial receptivity-related factors and used logistic regression to establish a predictive model for the probability of successful pregnancy. The results showed the nomogram prediction model with its value of area under the receiver operating curve (AUC) up to 0.949 for predicting pregnancy using age and ultrasonographic factors, including uterine peristalsis, uterine spiral artery, and ultrasound elastographic features (overview of the studies on ultrasound elastography in predicting the outcome of IUI is also listed in Table 2) [63]. By applying a pregnancy prediction model of ultrasonographic factors related to endometrial receptivity, clinicians can perform quantitative assessment and real-time screening of uterine conditions to provide optimal guidance, treatment, and management recommendations for infertility-related patients.

USE does not predict the outcome of IUI when used independently. However, using age and ultrasonographic factors, including SE, uterine motility, uterine spiral arteries, and ultrasound elastography features, can quantitatively estimate and predict pregnancy probability for clinicians. To date, studies using SWE to evaluate endometrial receptivity are lacking. Considering that SWE has the advantages of independent artificial pressure, more objectiveness, and more repeatability, the results of using SWE instead of SE to predict IUC deserve further exploration.

## Predicting preterm delivery

USE is an established method for evaluating cervical softening, predicting pre-term delivery and outcomes of labor induction [64–75]. In 2019, a meta-analysis including 1488 pregnant indicated that cervical USE is useful to PTD with a summary sensitivity of 0.84 [95% confidence interval (CI): 0.68, 0.93], a specificity of 0.82 (95% CI: 0.63, 0.93), a diagnostic odds ratio of 25 (95% CI: 7, 93), and AUC of USE being 0.90 (95% CI: 0.87–0.93) [76]. Induction of labor (IOL), a common practice in modern obstetrics, involves artificial labor stimulation before its spontaneous onset, and nearly one-quarter of all deliveries require IOL [70]. A group of studies concluded that SWE provides a promising method for predicting the efficacy of IOL. Strobel et al. included 41 full-term pregnancies who decided to accept IOL and SE, and assessments of the Bishop score were performed before and 3 h after IOL. They observed an association between strain patterns and SR values ​​at 3 h after IOL and a successful IOL (*p* = 0.0343 and *p* = 0.0342, respectively) that the results can well demonstrate after 48 h. This is the first study to demonstrate that cervical SE after the first application of prostaglandins helps predict the outcome of IOL [77]. Another study reported that measurement by SE is relatively reproducible with intra-observer reproducibility ICC 0.733 (95% CI 0.553–0.841) and inter-observer reproducibility ICC 0.801 (95% CI 0.666–0.881) [78]. A comparison of SWE and Bishop score was done in the Lu et al.’s study (*n* = 475), and outcome prediction models using inner cervical E and cervical length had increased AUC compared with models using the Bishop score (0.888 vs. 0.819, *p* = 0.009) [79]. Models based on SWE and cervical length had higher predictive accuracy than models based on the Bishop score.

If a single or combined biomarker is found in predicting PTB or IOL, it could reduce hospital costs and limit treatment [66]. Various approaches have been reported in the literature to improve the application of USE in obstetrics. Studies have shown that SE can qualitatively detect the elasticity of the cervix when using reference materials, but the application of this technique in cervical disease has not been studied [80]. Hamza et al. sought to combine lower uterine segment (LUS) thickness and SE to predict successful IOL within 24 h and intervals to onset of labor. However, LUS thickness and strain values ​​were not significant for predicting a successful IOL [81]. The tissue structure of the placenta (necrosis, inflammation, and possibly histological changes) can lead to preterm delivery [27]. When measured by SE, placental strain ratio (PSR) was inversely correlated with gestational age at birth, which is considered a valid predictor of PTD. Albayraket et al. analyzed the placenta and found that PSR has some promise in predicting PTD. This is because the fat-to-strain placenta ratio can be used to indicate PTD [82]. Tolunay et al. conducted a prospective study of threatened preterm labor (TPL) (*n* = 108) and measured PSR values. Multivariate logistic regression analysis showed that when the PSR value was 4.04, the sensitivity of short-term delivery time prediction was 77.78%, and the specificity was 87.04% [83]. SE may contribute to predict delivery time in TPL high-risk pregnancies. Therefore, we believe cervical elasticity combined with PSR should be beneficial for developing more effective preventive strategies for PTB.

5–18% of pregnant women are affected by PTD and it is the leading cause of neonatal death. This individualization of risk, both fetus and mother, leads to explicit management and treatment under a precision medicine approach [84]. Respiratory distress syndrome (RDS) occurs in 26 to 30 percent of preterm neonates before 34 weeks of gestation and 5 to 20 percent after 34 weeks of gestation [85]. Mottet et al. conducted a prospective case–control study including fetuses of uncomplicated pregnancies between 24 and 34 weeks of gestation (*n* = 55) and preterm-threatening pregnancies requiring corticosteroids (*n* = 48). SWE assessed fetal lung and liver elastography (LLE), and the results showed that there was no difference in LLE values between the two groups at “day 0,” but the LLE values decreased at “day 2” in the case group (0.2; 95% confidence interval: 0.07–0.34; *p* < 0.001). The repeatability and reproducibility of the measurement were calculated, and the results were acceptable [86, 87]. SWE could be considered a new non-invasive, reproducible tool for monitoring fetal lung development by assessing mechanical properties during pregnancy. In summary, we propose establishing a generalized risk prediction model including cervical elasticity, placental elasticity, and fetal LLE ratio to develop an evidence-based PTD risk assessment for clinical practice.

## Summary and future prospect

USE diagnosis is a promising diagnostic method, but its clinical application is limited due to instrument limitations and different elastography parameters; for example, SE can only provide semiquantitative results, while SWE can provide quantitative results. Given the advantages of SWE, the results are relatively operator-independent, while the shear wave is constant in the presence of a constant push pulse. We demonstrate that SWE is more suitable for clinical application and obstetricians are trained to use a phantom setup and an operating manual is achievable.

SWE has important application value in evaluating treatment response in uterine fibroids and adenomyosis. Whether USE can distinguish uterine fibroids from adenomyosis and whether the changes in adenomyosis are stiffer or softer than normal myometrial tissue remain controversial. Since the most generally accepted theory is that the disease develops through an alteration or absence of the JZ that causes the endometrial basal muscle to grow downward and invaginate into the myometrium, we hypothesized that measuring the SWV of the JZ could improve the accuracy of SWE in differentiating adenomyosis. This may provide new insights and potential therapeutic target strategies for the clinical strategies in the management of adenomyosis.

USE can significantly improve the diagnostic specificity of cervical cancer, and it is also useful for assessing infiltration the depth and stage of cervical cancer. In tumor tissues, stiffness is directly related to tumor development, invasion, metastasis, and chemoradiotherapy resistance; therefore, more research can focus on using USE to predict cervical cancer chemoradiotherapy treatment response. Moreover, the clinical importance of assessing the cervix after cervical conization is evident in most patients with CIN who are of childbearing age and wish to preserve fertility. Since algorithmically processed USE images combined with HPV detection have a better diagnosis of CIN, we presumed that studying the elastic properties of the cervix after cervical conization by this new technique has a great potential to predict future pregnancies. In addition, USE is useful for assessing cervical softening and then predicting premature delivery outcomes. Most studies were single-center studies, and further larger studies are needed. Simultaneous assessment of cervical elasticity, placental elasticity, and fetal lung maturity by SWE may predict preterm birth and neonatal respiratory complications for definitive management and treatment in a precision medicine approach.

For the foreseeable future, research into endometrial properties through USE will continue to focus on establishing the relationship between endometrial stiffness and fertility. With the application of SWE and the establishment of models to predict fertilization and pregnancy using age, uterine motility, uterine spiral arteries, and SWE characteristics, the clinical application of USE, especially in the field of infertility, will be significantly enhanced.

## Conclusions

Uterine stiffness is one of the important mechanical parameters, and some pathological processes may manifest as changes in the elasticity of uterine tissue. We believe that USE, especially shear wave elastography, may serve as a potential means to assess tissue stiffness, thereby improving the diagnosis and treatment of adenomyosis, fibroids, endometrial lesions, cervical cancer, and precise management of preterm birth and intrauterine insemination monitoring.

## Supplementary Information

**Additional file 1**. References for table 1 and table 2.

## Data Availability

Not applicable.

## Abbreviations

AEH: Atypical endometrial hyperplasia
AEH: Atypical endometrial hyperplasia
AM: Adenomyosis
ARFI: Acoustic radiation force impulse
AUC: Area under the receiver operating curve
B/A ratio: Ratio of mean elasticity of the endometrium to adjacent myometrium
CC: Cervical tumor
CI: Confidence interval
CIN: Cervical intraepithelial neoplasia
E max: Young’s modulus maximum
E mean: Young’s modulus mean
E/M ratio: Elasticity ratio about endometrial lesion to myometrium ratio
E/M ratio: Ratio of mean elasticity of the endometrial lesion to myometrial elasticity
EC: Endometrial carcinoma
GnRHa: GnRH agonists
HPV: Human papillomavirus
IOL: Induction of labor
IUI: Intrauterine insemination
IVF: In vitro fertilization
JZ: Uterus junctional zone
LLE: Lung-to-liver elastography
LUS: Lower uterine segment
MRE: Magnetic resonance elastography
MRI ADC: Magnetic resonance imaging apparent diffusion coefficient values
NM: Normal myometrium
PSR: Placental strain ratio
PTD: Predict preterm delivery
RDS: Respiratory distress syndrome
ROI: Region of interest
SE: Strain elastography
SR max: Strain ratio maximum
SR mean: Strain ratio mean
SR min: Strain ratio minimum
SR: Strain ratio
SWE: Shear wave elastography, Cs shear wave speed
SWI: Shear wave imaging
TE: Transient elastography
TPL: Threatened preterm labor
UAE: Uterine artery embolization
UF: Uterine fibroids
UF: Uterine fibroids
USE: Ultrasound elastography
WG: Weeks of gestation
