# Supplementary material for: Advances in the clinical application of ultrasound elastography in uterine imaging
Source: Insights Imaging. 2022 Sep 4;13:141. doi: 10.1186/s13244-022-01274-9 (PMC9440970; doi:10.1186/s13244-022-01274-9)
Supplement: Supplementary file 1 — Additional file 1. References for table 1 and table 2. [file 13244_2022_1274_MOESM1_ESM.pdf]

ELECTRONIC SUPPLEMENTARY MATERIAL

Advances in the clinical application of ultrasound elastography in uterine imaging

References for table 1

1.Manchanda S, Vora Z, Sharma R et al. (2019) Quantitative Sonoelastographic Assessment of the Normal Uterus Using Shear Wave Elastography: An Initial Experience Journal of ultrasound in medicine : official journal of the American Institute of Ultrasound in Medicine 38:3183-3189

2.Soliman AA, Wojcinski S, Degenhardt F (2015) Ultrasonographic examination of the endometrium and myometrium using acoustic radiation force impulse (ARFI) imaging technology: An initial experience with a new method Clinical hemorheology and microcirculation 59:235-243

3.Pongpunprut S, Panburana P, Wibulpolprasert P et al. (2022) A Comparison of Shear Wave Elastography between Normal Myometrium, Uterine Fibroids, and Adenomyosis: A Cross-Sectional Study International journal of fertility & sterility 16:49-54

4.Görgülü FF, Okçu NT (2021) Which imaging method is better for the differentiation of adenomyosis and uterine fibroids? Journal of gynecology obstetrics and human reproduction 50:102002

5.Zhang Y, Yan Y, Yang Y (2019) Study on value of ultrasonic elastography in diagnosis of clinical staging of cervical cancer and efficacy evaluation of radiotherapy Oncology letters 17:4901-4906

6.Bildaci TB, Cevik H, Yilmaz B, Desteli GA (2018) Value of in vitro acoustic radiation force impulse application on uterine adenomyosis Journal of medical ultrasonics (2001) 45:425-430

7.Stoelinga B, Hehenkamp WJK, Nieuwenhuis LL et al. (2018) Accuracy and Reproducibility of Sonoelastography for the Assessment of Fibroids and Adenomyosis, with Magnetic Resonance Imaging as Reference Standard Ultrasound in medicine & biology 44:1654-1663

8. Liu X, Ding D, Ren Y, Guo SW (2018) Transvaginal Elastosonography as an Imaging Technique for Diagnosing Adenomyosis Reproductive sciences (Thousand Oaks, Calif) 25:498-514
9. Frank ML, Schäfer SD, Möllers M et al. (2016) Importance of Transvaginal Elastography in the Diagnosis of Uterine Fibroids and Adenomyosis Ultraschall in der Medizin (Stuttgart, Germany : 1980) 37:373-378
10. Samanci C, Önal Y (2020) Shearwave elastographic evaluation of uterine leiomyomas after uterine artery embolization: preliminary results Turkish journal of medical sciences 50:426-432
11. Xie M, Yu H, Zhang X, Wang W, Ren Y (2019) Elasticity of adenomyosis is increased after GnRHa therapy and is associated with spontaneous pregnancy in infertile patents Journal of gynecology obstetrics and human reproduction 48:849-853

References for table 2

1. Vora Z, Manchanda S, Sharma R et al. (2022) Transvaginal Shear Wave Elastography for Assessment of Endometrial and Subendometrial Pathologies: A Prospective Pilot Study Journal of ultrasound in medicine : official journal of the American Institute of Ultrasound in Medicine 41:61-70
2. Ma H, Yang Z, Wang Y et al. (2021) The Value of Shear Wave Elastography in Predicting the Risk of Endometrial Cancer and Atypical Endometrial Hyperplasia Journal of ultrasound in medicine : official journal of the American Institute of Ultrasound in Medicine 40:2441-2448
3. Du YY, Yan XJ, Guo YJ et al. (2021) Transvaginal Real-Time Shear Wave Elastography in the Diagnosis of Endometrial Lesions International journal of general medicine 14:2849-2856
4. Gultekin IB, Imamoglu GI, Turgal M et al. (2016) Elastosonographic evaluation of patients with a sonographic finding of thickened endometrium European journal of obstetrics, gynecology, and reproductive biology 198:105-109
5. Czuczwar P, Wozniak S, Szkodziak P et al. (2016) Elastography Improves the Diagnostic

Accuracy of Sonography in Differentiating Endometrial Polyps and Submucosal Fibroids Journal of ultrasound in medicine : official journal of the American Institute of Ultrasound in Medicine 35:2389-2395

6.Kabukçu C, Çabuş Ü, Öztekin Ö, Fenkçi V (2021) The strain rate of endometrium measured by real-time sonoelastography as a predictive marker for pregnancy in gonadotropin stimulated intrauterine insemination cycles The journal of obstetrics and gynaecology research 47:3561-3570

7.Shui X, Yu C, Li J, Jiao Y (2021) Development and validation of a pregnancy prediction model based on ultrasonographic features related to endometrial receptivity American journal of translational research 13:6156-6165

8.Swierkowski-Blanchard N, Boitrelle F, Alter L et al. (2017) Uterine contractility and elastography as prognostic factors for pregnancy after intrauterine insemination Fertility and sterility 107:961-968.e963
